# Flute-type porous carbon derived from soybean shells for high-performance all-solid-state symmetric supercapacitors

**DOI:** 10.1039/d2ra06216j

**Published:** 2022-11-14

**Authors:** Yan Wu, Yan Wang, Le Pan, Xiao Ran Wu

**Affiliations:** School of Chemistry and Chemical Engineering, Huangshan University Huangshan 245041 PR China 17712965571@163.com

## Abstract

Flute-type porous carbon was successfully prepared from soybean shells through convenient methods. The influence of mass ratio on the structure and electrochemical performance of porous carbon obtained from soybean shells was investigated in detail. The obtained porous carbon exhibited a micro-tube morphology structure with a specific surface area of 2802 m^2^ g^−1^, pore volume of 1.36 cm^3^ g^−1^, and appropriate pore size distribution. The porous carbon showed good electrochemical properties as an electrode material for supercapacitors. The optimal porous carbon SSAC4 exhibited high specific capacitance of 465 F g^−1^ (1 A g^−1^) and 287 F g^−1^ (20 A g^−1^) in a three-electrode system with 6 M KOH electrolyte. In addition, the as-assembled SSAC4-based all-solid-state supercapacitors delivered a high specific capacitance of 294 F g^−1^ at 0.1 A g^−1^ and excellent cycling stability of 86.2% after 10 000 cycles at 5 A g^−1^.

## Abstract 2

Flute type porous carbon is successfully derived from soybean shell through a convenient method. The porous carbon shows good electrochemical properties as an electrode material for supercapacitors.

## Graphical abstract

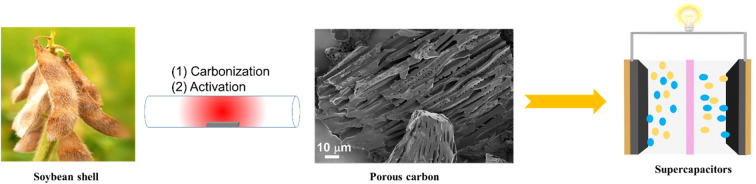

## 1. Introduction

Recently, the rapid consumption of fossil fuels and increasing environmental pollution have prompted the development of renewable, sustainable, and environment-friendly energy storage systems. New energy resources, such as solar, tide power, wind, and supercapacitors, are being developed.^1^ Supercapacitors are being considered as ideal energy storage devices due to their long cycle life, rapid charge–discharge rate, high power density, safety, and environment.^2–4^ Therefore, supercapacitors are applied to various applications in hybrid electric vehicles, portable devices, industrial power, and other fields.

Supercapacitors are classified into electrical double-layer capacitors (EDLCs) and pseudocapacitors according to their different energy storage mechanisms.^5^ The pseudocapacitors store energy mainly based on redox reactions on/near the surface of the metal oxide electrode, therefore, they usually have a larger specific capacitance.^6^ However, the practical application of pseudocapacitors is greatly limited due to poor cycle stability, high price, and low electrical conductivity. On the contrary, the EDLC store energy by the electric double-layer capacitance formed between the electrode/electrolyte; thus they have excellent stability and good rate performance.^7–9^ The electrolytes of supercapacitors include liquid electrolytes and solid-state electrolytes. Compared with solid-state electrolytes, liquid electrolytes face the problems of potential leakage and consequent internal short-circuits.^10^ All-solid-state supercapacitors are widely applied in the area of portable and wearable electronics in our daily lives owing to their intrinsic properties such as enhanced safety, flexibility, and thin-film forming ability.^11,12^

As is known, the electrochemical performances of the electrode materials are determined not only by the specific surface area but also by the pore structure and surface chemistry. Among various electrode materials, porous carbon is usually an electrode material for supercapacitors due to its low cost, high specific surface area, well-developed pore structure, and excellent chemical stability.^13–15^ Biomass is the most promising precursor for prepared porous carbon *via* the activation method, hard template method, hydrothermal method combined with the activation method, soft template method, and so on.^16–18^ For example, Song *et al.*^19^ synthesized a series of porous carbon from mung bean husks through pyrolysis and hydrothermal methods combined with KOH activation. In addition, the obtained porous carbon possessed good electrochemical performance when used as the electrode material. Hollow-tubular porous carbon with a high specific surface area of 1508 m^2^ g^−1^ and an obvious mesoporous structure was synthesized from natural biomass cotton through a facile carbonization and KOH–KNO_3_ activation process.^20^ The hollow-tubular porous carbon exhibits high specific capacitance (278 F g^−1^ at 1 A g^−1^) and excellent rate capability (208 F g^−1^ at 100 A g^−1^). Two-dimensional hierarchical porous carbon sheets (FHPCs) were derived from coal tar pitch using nano-MgO sheets as a template. The FHPCs showed good electrochemical performance as an electrode material for supercapacitors, such as a high capacitance of 290 F g^−1^ at 1 A g^−1^ and an excellent rate capability with 86% retention rate in 6 M KOH electrolyte.^21^

The structural properties of porous carbon are not only affected by the preparation process but also affected by the raw materials.^19,22–24^ Among various carbon precursors, biomass become the most promising raw material owing to its merits of being renewable, environmentally friendly, low cost, and unique structure.^19^ As an important crop, soybean is widely planted in China. Therefore, a large number of soybean shells are generated every year, but most of them are sent to landfill or are directly incinerated leading to environmental pollution and waste of resources. It is of great significance to transform soybean shells into high-value-added porous carbon through appropriate process treatment. In this work, the porous carbon was obtained from the soybean shell through carbonization followed by KOH activation. The porous carbon used as a supercapacitors electrode shows good electrochemical performances in both 6 M KOH electrolyte supercapacitors and all-solid-state supercapacitors.

## 2. Experimental section

### 2.1 Sample preparation

The original soybean shell was collected from Xuzhou, Jiangsu, which was washed with ethanol and deionized water and then dried in an oven overnight. HCl, KOH, and poly(vinyl alcohol) (PVA) were purchased from Sinopharm Co. Ltd (China). In a typical case, a 4 g of soybean shell (power) was heated to 400 °C for 2 h at the heating rate of 10 °C min^−1^ under an Ar atmosphere. After carbonization, the carbonized sample was mixed with KOH at different mass ratios and then fully ground in an agate mortar. The mixture was placed in a tube furnace and the temperature was increased to 700 °C at a heating rate of 10 °C min^−1^ and maintained for 2 h. Finally, the activated sample was washed in 2 M HCl solution and deionized water several times. The washed sample was dried at 120 °C overnight. The as-prepared products were named SSAC*n*, where *n* (*n* = 2, 3, 4, and 5) stands for the mass ratio of KOH to the carbonized product.

### 2.2 Characterization

The morphology and microstructure of the obtained samples were studied by scanning electron microscopy (SEM, Zeiss Gemini 500) and transmission electron microscopy (TEM, JEOL-2010). The crystal structure was studied using a powder X-ray diffractometer with Cu Kα radiation (XRD, Bruker D8 Advance, *λ* = 1.5418 Å, 40 kV, 40 mA). Raman spectra were recorded on a Raman spectrometer with a laser wavelength of 532 nm (Bruker, Senterra). The surface chemical composition of samples was studied using X-ray photoelectron spectroscopy (XPS, Thermo Fisher, ESCALAB 250Xi, Al Kα radiation). The specific surface area and porosity of samples were measured using a nitrogen adsorption–desorption (Gold APP V-Sorb 4800TP) instrument. The nitrogen adsorption–desorption isotherms were collected at 77 K and the samples were pretreatment in a vacuum oven at 300 °C for 10 h. The specific surface area was obtained from the Brunauer–Emmette–Teller (BET) equation, and the density functional theory (DFT) method was used to obtain the pore size distribution. In order to evaluate the thermal degradation behaviors, thermal gravimetric (TG) analysis coupled with derivative thermogravimetry (DTG) analysis was conducted using a Mettler Toledo TGA/DSC1 analyzer in Ar at a heating rate of 5 °C min.

### 2.3 Electrochemical measurements in a three-electrode system

The electrochemical performances of the single electrode were studied on a standard three-electrode system in 6 M KOH. The working electrode was prepared by mixing the active material, acetylene black and polytetrafluoroethylene (PTFE) with a mass ratio of 85 : 10 : 5. The mixture was coated onto the nickel foam followed by drying at 80 °C, overnight, in a vacuum oven. Hg/HgO and a Pt foil were used as the reference and counter electrodes, respectively. The cyclic voltammeter (CV) and electrochemical impedance spectroscopy (EIS) studied were performed on an Ivium Vertex electrochemical workstation. The galvanostatic charge–discharge (GCD) was studied using the NEWARE battery test system. The specific capacitance (*C*_sp_) in a three-electrode system was calculated from the GCD curves using eqn (1).1
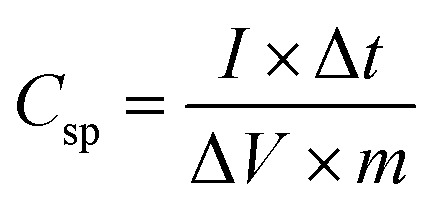
where *I* (mA), Δ*t* (s), Δ*V* (mV), and *m* (g) are the discharging current, discharging time, and discharging potential range excluding the IR drop and mass of active material on the electrode, respectively.

### 2.4 Fabrication of all-solid-state supercapacitors

2 g of PVA was dissolved in 20 mL of deionized water under vigorous stirring at 90 °C for 1 h to form a transparent PVA solution. Then, 10 mL of 6 M KOH solution was slowly added to the PVA solution using a peristaltic pump under vigorous stirring. Finally, the mixture solution was poured into a Petri dish and allowed to solidify. An all-solid-state symmetric supercapacitor was fabricated from a PVA/KOH gel film between the two sheets of SSAC*n*. The specific capacitance of a single electrode was calculated according to the following eqn (2).2
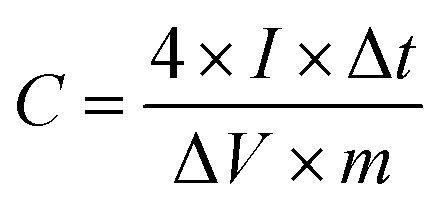
where *I* (mA), Δ*t* (s), and Δ*V* (mV) have the same meaning as described for the terms in eqn (1), *m* (g) is the total mass of active material in the two working electrodes. The energy density (*E*, W h kg^−1^) and power density (*P*, W kg^−1^) of symmetrical supercapacitors systems were calculated by using eqn (3) and (4).3
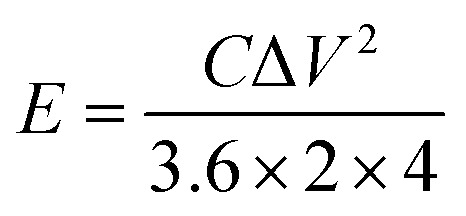
4
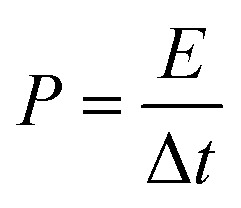


## 3. Results and discussion

### 3.1 Material characterization

**Fig. 1:**
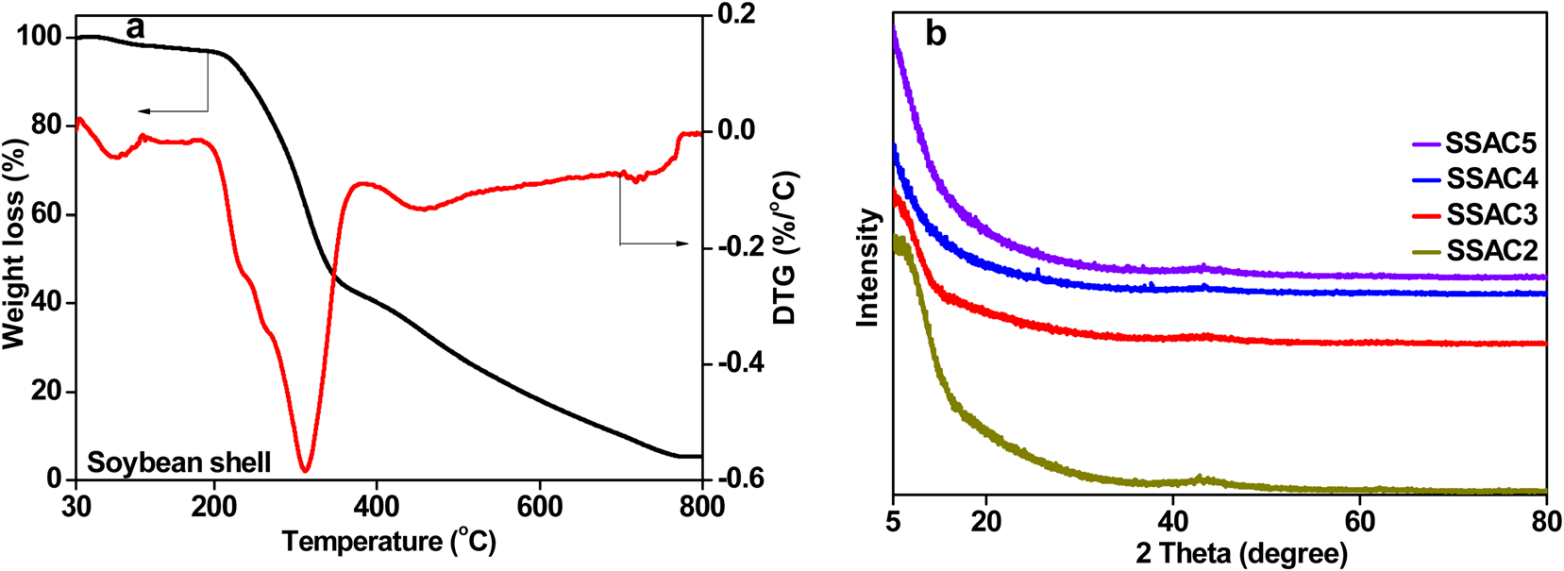
TG and DTG curves of soybean shell (a) and XRD spectra of SSAC*n* samples (b).

The thermal degradation behavior of soybean shells, including the maximum degradation rate temperature and mass loss percentage, was studied using the thermal gravimetric analysis, and the results are shown in Fig. 1a. The weight loss at the temperature below 100 °C is due to the dehydration of the sample, reflected in the DTG curve as a small peak. The maximum weight loss in the temperature range of 180–384 °C is owing to the decompositions of cellulose, hemicellulose, and lignin. The corresponding peak at 312 °C can be observed in the DTG curve. The unstable carbon edges with the oxygen-containing group decomposed cause a little weight loss when the temperature is from 500 to 800 °C.^25^ Therefore, in this work, the soybean shell was carbonized at 400 °C, followed by activation with KOH.

**Fig. 2:**
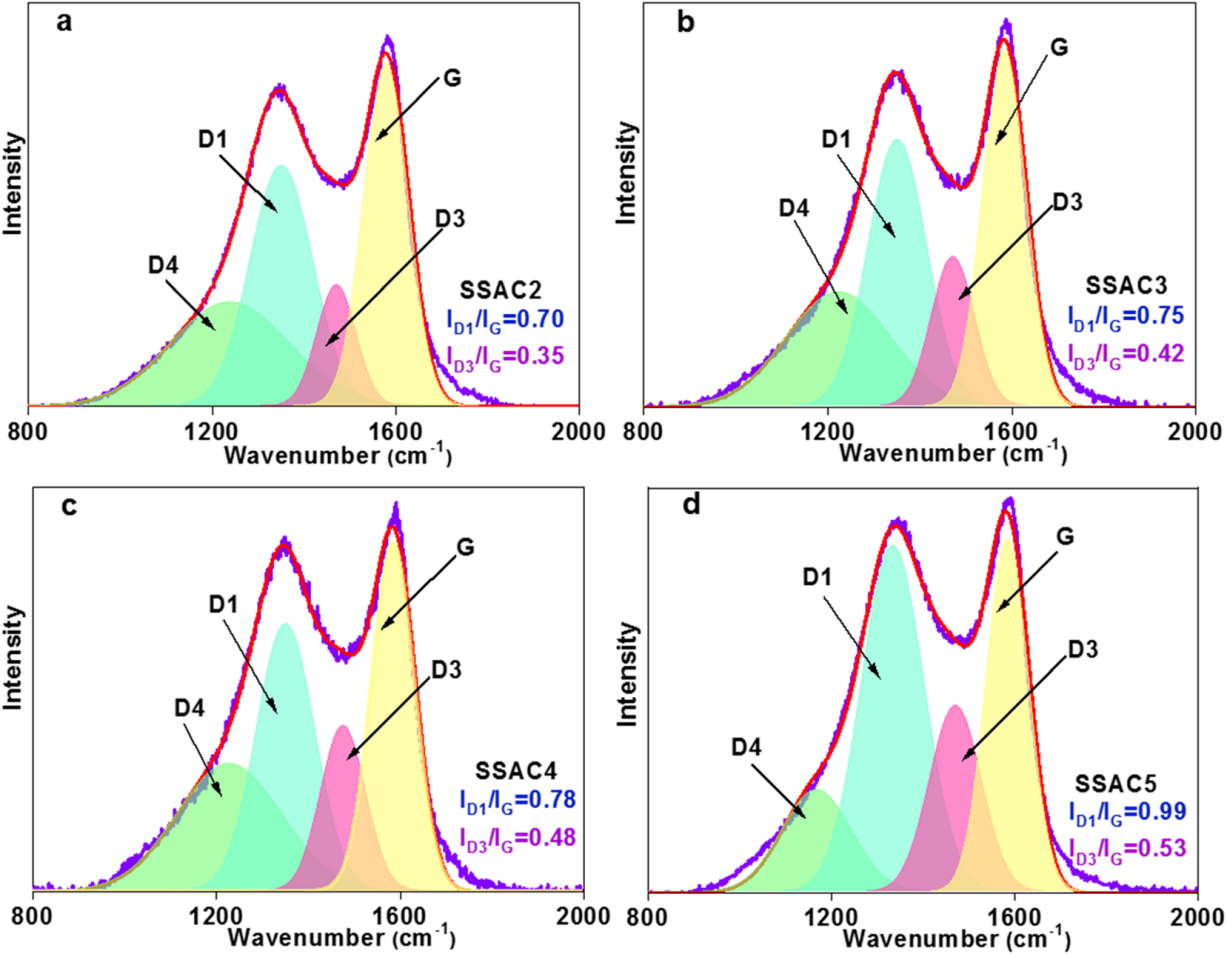
Raman spectra of SSAC*n* (a)–(d).

The XRD patterns of SSAC*n* are shown in Fig. 1b, where two not obvious peaks were seen at about 25° (002) and 43° (100), revealing the amorphous structure of SSAC*n*. The low intensity of the (002) peak is owing to the formation of more defects in the KOH activation process.^26^ In addition, the SSAC*n* possesses high intensity in the small angle region (2*θ* < 20°), demonstrating the existence of high-density of micropores.^27^ The crystallinity of the SSAC*n* also can be characterized using Raman spectroscopy. The Raman spectrum can be resolved into four peaks (Fig. 2) at around 1352 cm^−1^ (D1-band), 1480 (D3-band), 1240 cm^−1^ (D4-band) and 1581 cm^−1^ (G-band), where D1-band belongs to the disordered structure, D3-band represents the amorphous carbon structure, D4-band is related to the polyenes or ionic impurities and G-band refers to the graphite structure.^28,29^ The value of *I*_D1_/*I*_G_ represents the graphitization degree of the SSAC*n*. With the activation ratio from 2 : 1 to 5 : 1, the value of *I*_D1_/*I*_G_ increases from 0.70 to 0.99, while the *I*_D3_/*I*_G_ also increases from 0.35 to 0.53, indicating that the order degree of the SSAC*n* decreased. In other words, more defects and disordered carbon lattices are generated in excessive KOH. The porosity is formed due to KOH etching of the carbon materials based on the following reactions:

5 6KOH + 2C → 2K + 3H_2_ + 2K_2_CO_3_

6 K_2_CO_3_ → K_2_O + CO_2_

7 K_2_CO_3_ + 2C → 2K + 3CO

8 K_2_O + 2C → 2K + CO

With the increase of KOH content, more KOH takes part in the reaction, resulting in more pore formation and even some pore collapse. Therefore, the specific surface area of SSAC*n* increases first and then decreases with the increasing activation ratio (Table 1).

**Table 1:** Porosity characteristics of PCs prepared with different activation temperatures and KOH/hydrochar ratios

Sample	*S*_BET_ a (m^2^ g^−1^)	*V*_t_ b (cm^3^ g^−1^)	*S*_micro_ c (m^2^ g^−1^)	*V*_micro_ c (cm^3^ g^−1^)	*S*_ext_ d (m^2^ g^−1^)	*V*_ext_ d (cm^3^ g^−1^)	*D*_ave_ e (nm)
SSAC2	2071	0.93	1960	0.80	111	0.13	1.80
SSAC3	2498	1.17	2351	1.01	147	0.16	1.88
SSAC4	2802	1.36	2619	1.15	183	0.21	1.94
SSAC5	2365	1.30	2124	1.04	241	0.26	2.19

a *S*_BET_: specific surface area from multiple BET method.

b *V*_t_: total pore volume at *P*/*P*_0_ = 0.99.

c *S*_micro_, *V*_micro_: micropore surface area and micropore volume from the *t*-plot method.

d *S*_ext_, *V*_ext_: difference of *S*_BET_ and *S*_micro_ and total pore volume and *V*_micro_, respectively.

e *D*_ave_: average pore diameter.

**Fig. 3:**
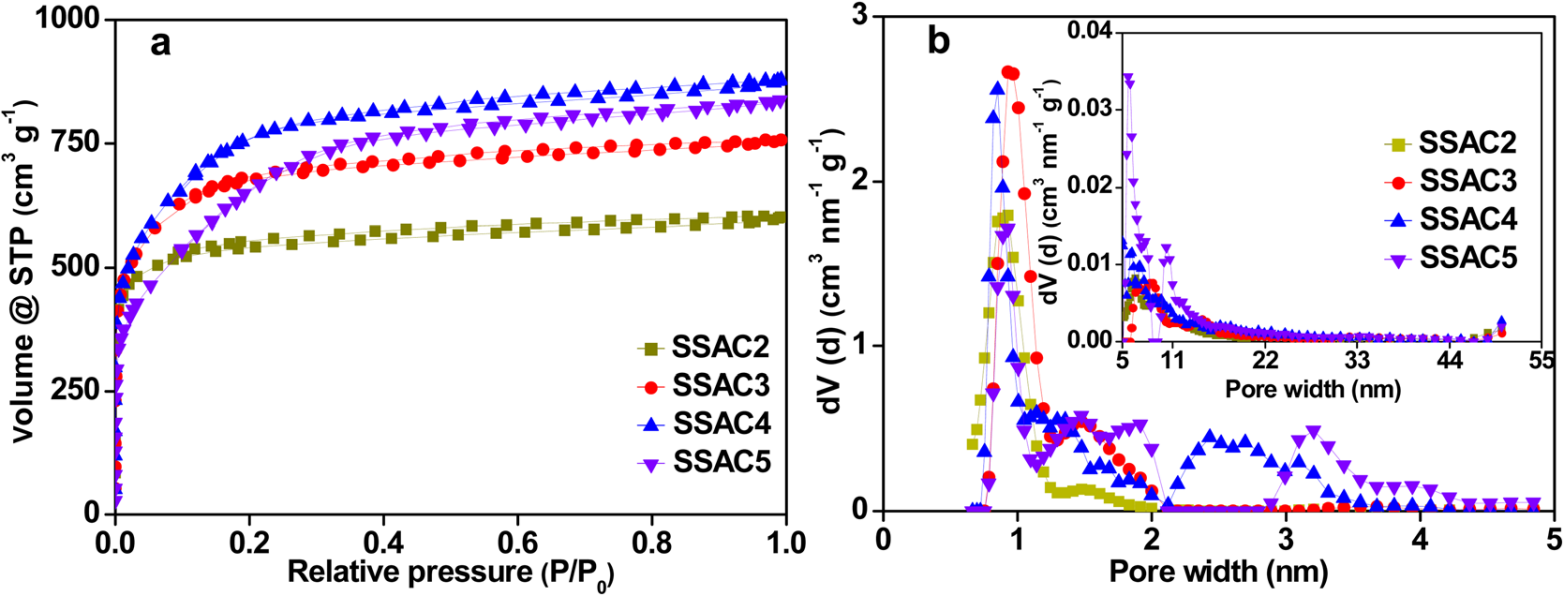
N_2_ adsorption–desorption isotherms (a) and pore size distribution of SSAC*n* (b).

As shown in Fig. 3a, the isotherms of SSAC*n* belong to a combination of type I and IV. The adsorption amount rises sharply at low relative pressure, indicating that SSAC*n* possesses high-density micropores. There is a hysteresis loop at the relative pressure from 0.3 to 0.9, demonstrating the presence of some mesopores in SSAC*n*. The detailed porosity parameters are presented in Table 1. The specific surface area, micropore surface area, and micropore volume increase with the activation ratio from 2 to 4, indicating that the formation of new pores and previously inaccessible pores possess the dominant reaction in a low activation ratio. When the activation ratio is above 4, the specific surface area decreases while the *S*_ext_ and *V*_ext_ still increase, suggesting that the expansion of the existing pores is the dominant factor in the high activation ratio. A similar result was also seen in the pore size distribution. As presented in Fig. 3b, the pores of SSAC*n* display a hierarchical pore structure composed of micropores and mesopores. The pore size of SSAC*n* is mainly centered at 0.83–0.96 nm (small micropores), 1.16–1.93 nm (large micropores), and 2.17–4.24 nm (mesopores). It is worth noting that some small micropores transform into large micropores or mesopores with an increase in the activation ratio, indicating that the pore size can be controlled by adjusting the activation ratio. The appropriate pore size is beneficial to enhance the charge storage capacity and the ion-transport capacity even at the high current density.

**Fig. 4:**
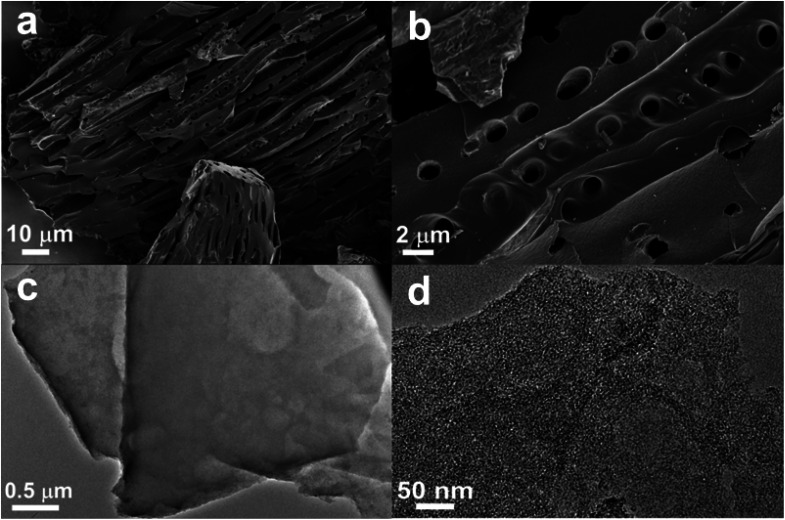
SEM (a) and (b) and TEM (c) and (d) images of SSAC4.

As Fig. 4a and b show, SEM images of SSAC4 show a micro-tube morphology with a pore in the surface, forming a flute-like structure. The hollow microtube can reduce the transmission resistance of electrolytes and improve the electrochemical performance when the samples are used as the electrode material. In addition, it is easily observed that some pore structures on the surface of SSAC4 can store more electrolyte ions. The morphology and structure of SSAC4 were further measured by TEM and the results are shown in Fig. 4c and d. Bright overlapping holes can be clearly observed in Fig. 4c, indicating that a number of macropores exist in SSAC4. The high-magnification TEM image (Fig. 4d) indicates an abundance of micropores and a small number of microcrystalline regions exist in SSAC4, indicating that the as-prepared sample is mainly amorphous carbon.^19^

**Fig. 5:**
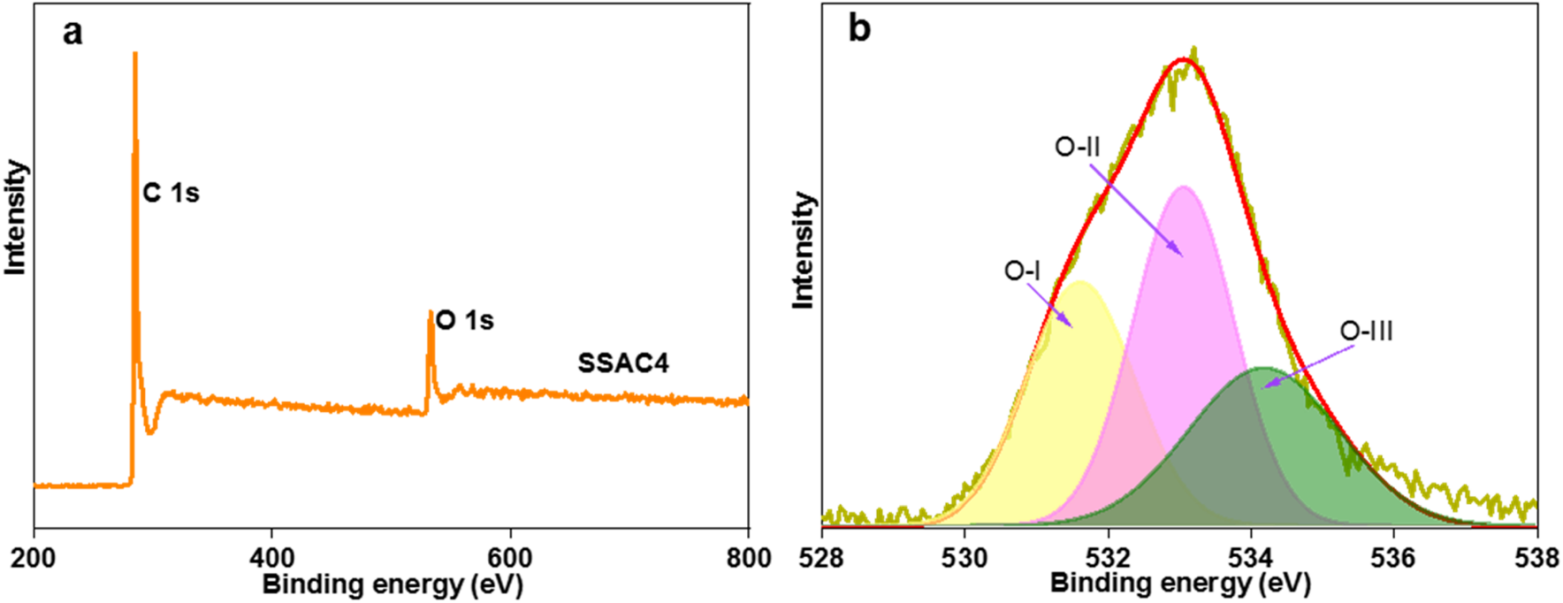
XPS survey spectra (a) and high-resolution spectra of O 1s (b) for SSAC4.

Furthermore, the surface chemical composition and states of the surface atoms of SSAC4 were analyzed by XPS. As depicted in Fig. 5a, there are two obvious peaks at binding energies of 285.1 eV and 532.5 eV and a small peak at 401.2 eV, which correspond to C 1s, O 1s, and N 1s, respectively. The corresponding contents of C 1s, O 1s and N 1s are 89.25 at%, 10.64 at% and 0.11 at%, respectively. N and O heteroatoms can not only improve the wettability, conductivity, and stability of the sample but also produce additional pseudocapacitance.^30,31^ In addition, as shown in Fig. 5b, the high-resolution O 1s can be divided into three peaks at 531.6, 533.1, and 534.2 eV, which are attributed to C=O, C–O–C/C–OH and –COOH, respectively.^32^ These oxygen-containing groups can not only enhance the wettability of electrode materials but also increase the specific capacitance *via* redox reactions.^27^

### 3.2 Electrochemical performance of the SSAC*n* electrodes in a three-electrode system

**Fig. 6:**
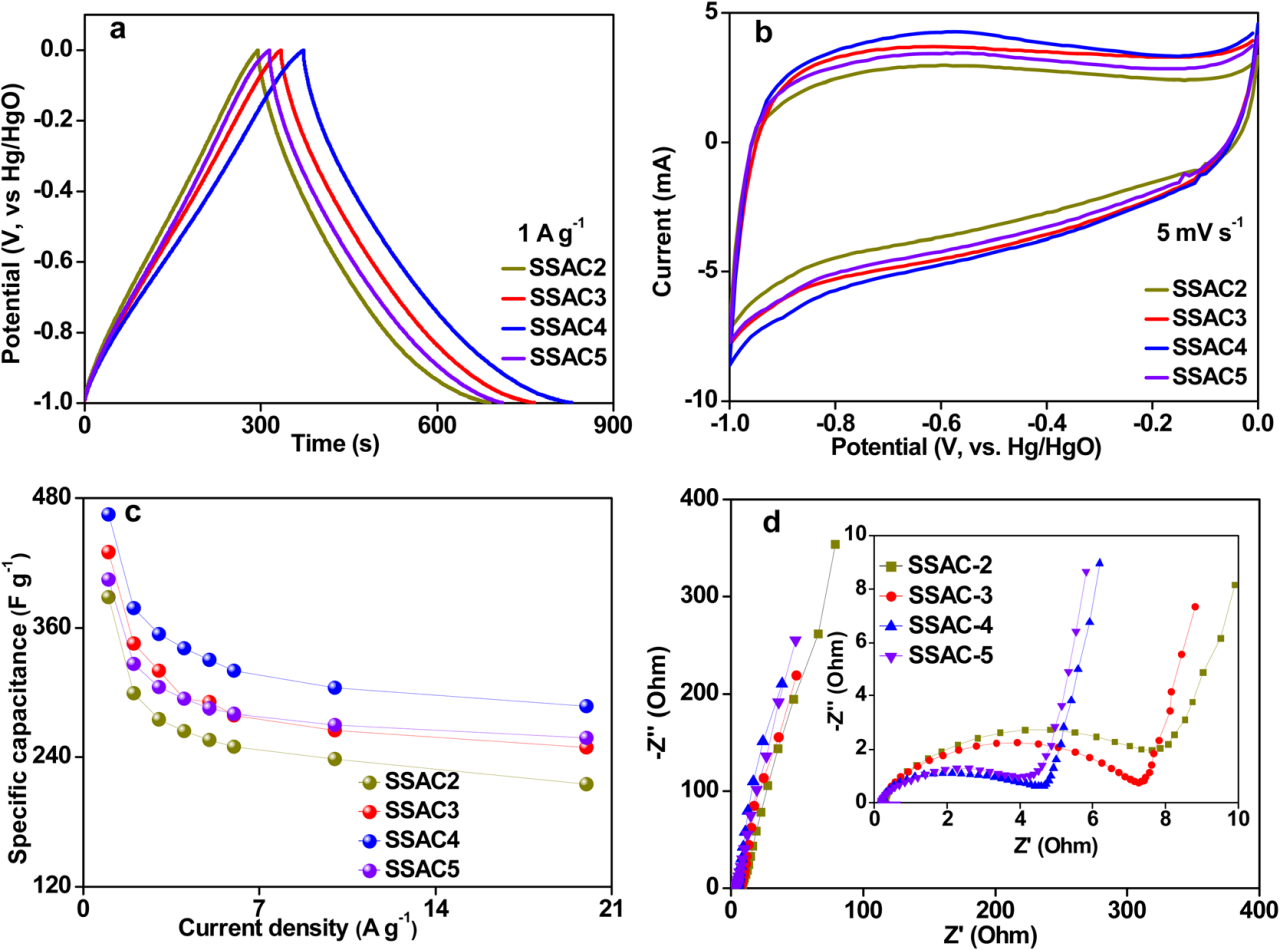
Electrochemical performance of SSAC*n* measured using a three-electrode system in 6 M KOH electrolyte. GCD curves at a current density of 1 A g^−1^ (a), CV curves at a scan rate of 5 mV s^−1^ (b), the *C*_sp_ of SSAC*n* at different current densities (c), and Nyquist plots (d).

The electrochemical performances of SSAC*n* were investigated using a three-electrode system with 6 M KOH as the electrolyte. As presented in Fig. 6a, GCD curves of SSAC*n* at a current density 1 A g^−1^ have a symmetrical triangle shape with a tiny distortion, suggesting that the specific capacitance of SSAC*n* is mainly derived from the double layer capacitance and a small amount of pseudocapacitance generated by oxygen group redox reactions. The CV curves (Fig. 6b) of SSAC*n* display a quasi-rectangular shape with a Faraday hump in the potential range from −0.9 V to −0.3 V, indicating that the specific capacitance of SSAC*n* comes from the combination of double-layer capacitance and pseudocapacitance, which shows good consistency with the results of GCD. The pseudocapacitance of SSAC*n* is the result of the Faraday redox reactions based on the following reactions:^33,34^

9

<svg xmlns="http://www.w3.org/2000/svg" version="1.0" width="10.400000pt" height="16.000000pt" viewBox="0 0 10.400000 16.000000" preserveAspectRatio="xMidYMid meet"><metadata>
Created by potrace 1.16, written by Peter Selinger 2001-2019
</metadata><g transform="translate(1.000000,15.000000) scale(0.011667,-0.011667)" fill="currentColor" stroke="none"><path d="M80 1160 l0 -40 40 0 40 0 0 -40 0 -40 40 0 40 0 0 -40 0 -40 40 0 40 0 0 -40 0 -40 40 0 40 0 0 -40 0 -40 40 0 40 0 0 -40 0 -40 40 0 40 0 0 -40 0 -40 40 0 40 0 0 80 0 80 -40 0 -40 0 0 40 0 40 -40 0 -40 0 0 40 0 40 -40 0 -40 0 0 40 0 40 -40 0 -40 0 0 40 0 40 -40 0 -40 0 0 40 0 40 -80 0 -80 0 0 -40z M560 520 l0 -40 -40 0 -40 0 0 -40 0 -40 -40 0 -40 0 0 -40 0 -40 -40 0 -40 0 0 -40 0 -40 -40 0 -40 0 0 -40 0 -40 -40 0 -40 0 0 -40 0 -40 -40 0 -40 0 0 -40 0 -40 80 0 80 0 0 40 0 40 40 0 40 0 0 40 0 40 40 0 40 0 0 40 0 40 40 0 40 0 0 40 0 40 40 0 40 0 0 40 0 40 40 0 40 0 0 80 0 80 -40 0 -40 0 0 -40z"/></g></svg>

C–OH ⇔ C=OH + H^+^ + e^*−*^

10 –COOH ⇔ –COO + H^+^ + e^*−*^

11 C=O + e^*−*^ ⇔ C–O^−^

In addition, the longer GCD curve of SSAC4 (Fig. 6a) compared to that of SSAC2, SSAC3, and SSAC5 demonstrates its higher specific capacitance, which is because SSAC4 possesses a high specific surface area and abundant hierarchical pore structure. The CV curve of SSAC4 displays the largest CV area, further indicating that SSAC4 possesses the largest specific capacitance. The specific capacitance of SSAC*n* at various current densities was calculated from GCD curves using eqn (1) and the results are presented in Fig. 6c. Significantly, the capacitance value of SSAC4 can reach up to 465 F g^−1^ at 1 A g^−1^, which is higher than that for the biomass-derived carbon material reported in recent literature (Table 2). As depicted in Fig. 6c, the specific capacitance of SSAC3 is higher than that of SSAC5 at low current density because the electrolyte ions have enough time to reach the electrode surface to form an electric double layer at a low current density and the high micro-specific surface area of SSAC3 can provide more active sites. While, the specific capacitance of SSAC5 is higher than that of SSAC3 at high current densities (Fig. 6c), which is because SSAC5 possesses more mesopores compared to SSAC3. Mesopores are beneficial for the rapid transport of electrolyte ions, resulting in enhancing the rate performance of SSAC5.

**Table 2:** Summary of the electrochemical parameters of the biomass-derived carbon electrodes

Precursor	Test system	Electrolyte	Current density (A g^−1^)	Specific capacitance (F g^−1^)	Ref.
Black locust (*Robinia pseudoacacia*) seed	3 E	6 M KOH	1	333	35
*Moringa oleifera* branches	3 E	6 M KOH	0.5	374	36
Agricultural wastes	3 E	6 M KOH	1	225	37
Cotton	3 E	6 M KOH	1	278	20
Mung bean husk	3 E	6 M KOH	1	390	19
Rape pollen	3 E	6 M KOH	1	361.6	38
Soybean shell	3 E	6 M KOH	1	465	This work

The electrochemical behavior of SSAC*n* was also investigated by the EIS and the results are presented in Fig. 6d. All the Nyquist plots show a nearly vertical line at the low frequency, suggesting the ideal capacitive performance of supercapacitors. The small intercept value of the real axis indicated that the SSAC*n* have lower ohmic resistance (*R*_s_) and the *R*_s_ values for SSAC2, SSAC3, SSAC4, and SSAC5 are 0.26, 0.24, 0.21, and 0.20, respectively. The semicircle diameter at the high frequencies indicates the response to the charge-transfer resistance (*R*_ct_) at the electrode/electrolyte interface.^39^ The large micropores and mesopores are favourable to the ions' transport, resulting in a decrease in the value of *R*_ct_ when the activation ratio is increased. Therefore, SSAC*n* derived from soybean shells used as the electrode material for supercapacitors have good electrochemical performance.

**Fig. 7:**
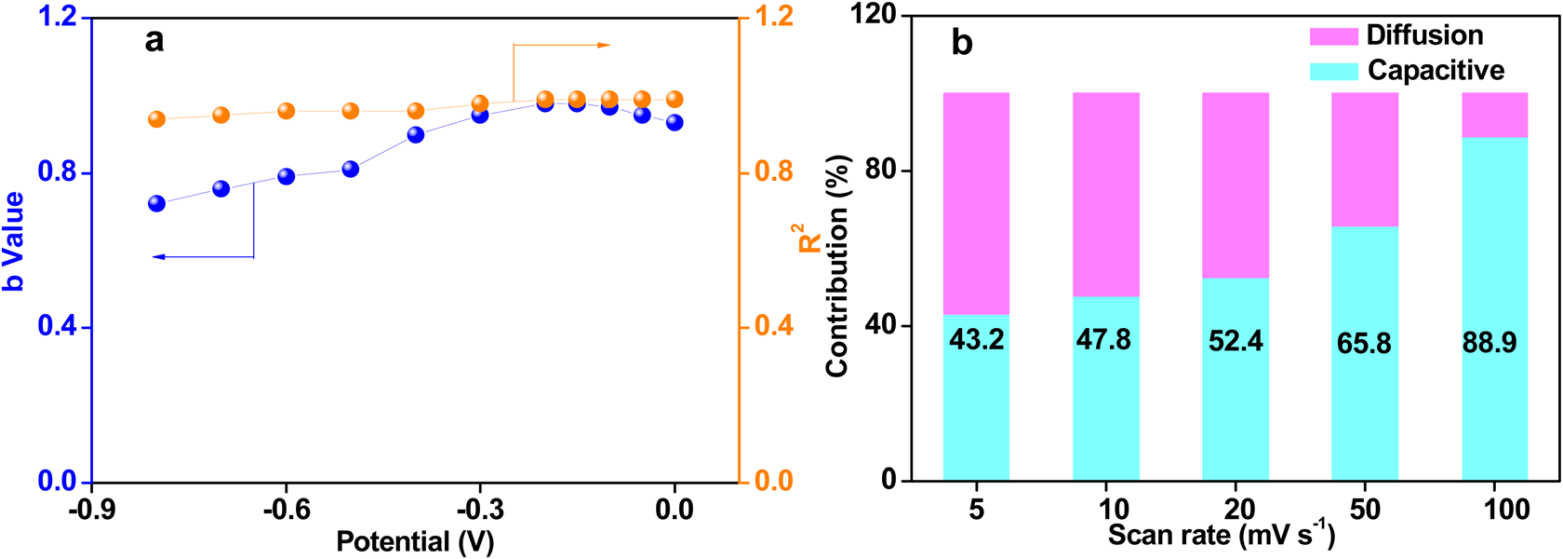
*b* values plotted against the potential of the supercapacitor and *R*^2^ values are the corresponding fitting variance (a) and columnar contribution map of surface control capacitance at different scan rates (b).

The capacitance of supercapacitors mainly includes surface-controlled capacitance and diffusion-controlled capacitance.^40^ The surface-controlled capacitance is composed of faradaic capacitance from the redox reaction on the surface and the electric double layer capacitance from the electrolyte ions adsorption–desorption, while the diffusion control capacitance primarily originates from the embedding of the electrolyte ions.^29,40^ The charge storage mechanism of supercapacitors can be calculated based on the following equation:

12 *I* = *av*^*b*^

where the measured current (*I*) at a fixed potential obeys a power law relationship with the potential sweep rate (*v*). Both a and *b* are constants and the value of *b* is usually in the range of 0.5–1.0. When *b* = 0.5, the charge storage mechanism of supercapacitors is dominant by the diffusion-controlled contribution. While the surface-controlled process is dominant when the *b* approaches 1.^41^ As shown in Fig. 7a, the value of *b* is over 0.75 with the potential range from −1.0 to 0 V and the fitting variance *R*^2^ is close to 1, suggesting that the surface-controlled contribution is dominant for charge storage of the SSAC4-derived electrodes. Dunn's equation was used to further analyze the capacitive contribution of the SSAC4 electrodes.

13 *I*(*v*) = *k*_1_*v* + *k*_2_*v*^1/2^

where *I*(*v*) corresponds to the current at a fixed potential, *k*_1_*v* and *k*_2_*v*^1/2^ are attributed to the surface and diffusion-controlled processes, respectively.^42^ At a scan rate of 100 mV s^−1^, the surface control capacitance for charge storage was as high as 88.9% (Fig. 7b). In addition, the contribution of the surface control capacitance was from 43.20% to 88.9% with the scan rate in the range from 5 to 100 mV s^−1^, indicating that the contribution of diffusion control capacitance to charge storage shows a rapid reduction with the increasing scan rate. Moreover, the dominant contribution from the surface control capacitance shows the good surface charge storage capability and the better rate performance of SSAC4.

### 3.3 Electrochemical performance of the all-solid-state symmetric supercapacitors

**Fig. 8:**
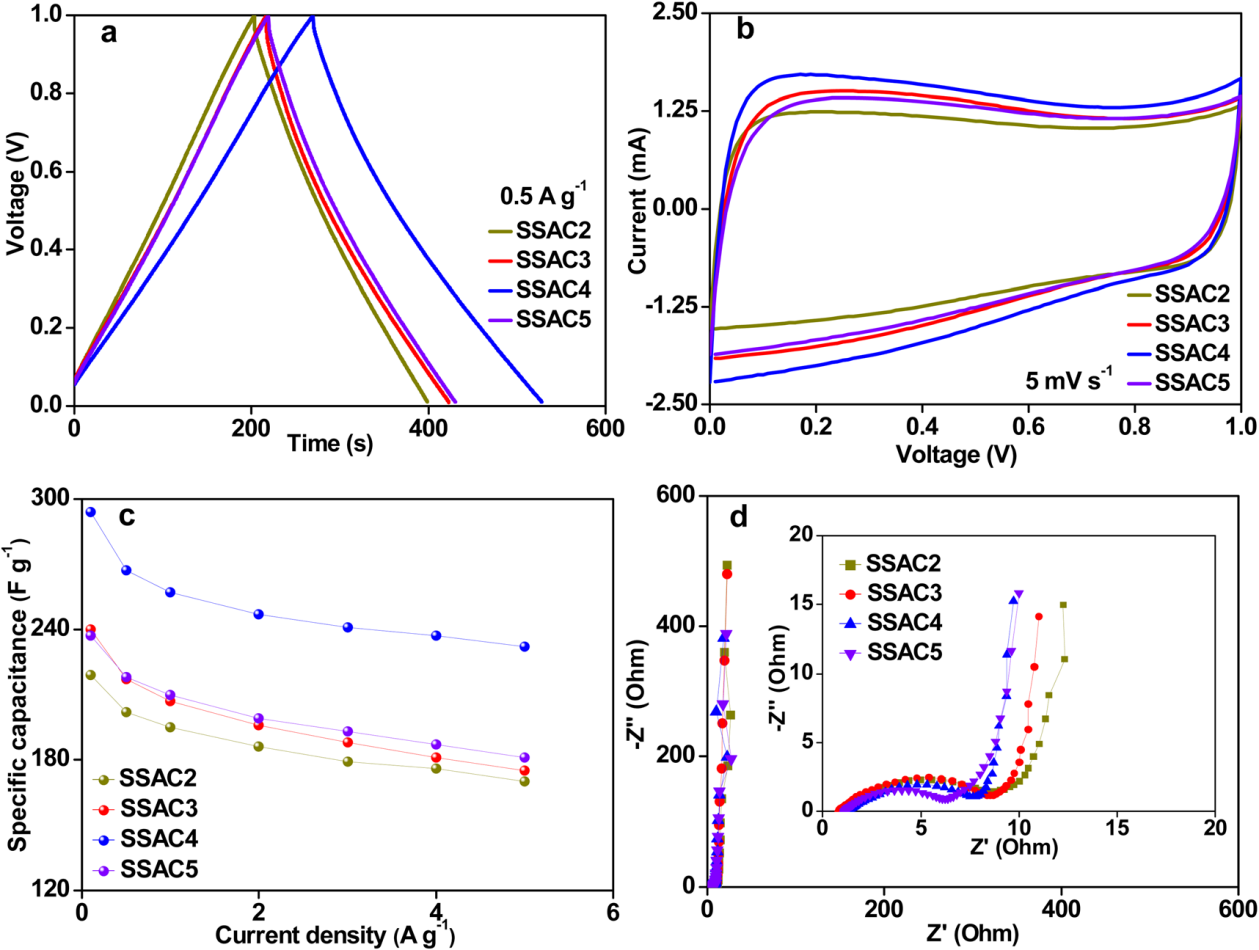
Electrochemical performance of SSAC*n* in the all-solid-state supercapacitors in PVA/KOH electrolyte. GCD curves at 0.5 A g^−1^ (a), CV curves at 5 mV s^−1^ (b), the specific capacitance at different current densities (c), and Nyquist plots (d).

In order to explore the practical application of the SSAC*n* electrode, the symmetric supercapacitor's performance based on the SSAC*n* electrode was investigated in PVA/KOH electrolyte. As plotted in Fig. 8a, the GCD curves of SSAC*n* obtained from the all-solid-state symmetric supercapacitors show quasi-symmetrical triangular shapes with limited voltage (IR) drop, implying the SSAC*n* possess good electrochemical reversibility. According to eqn (2), SSAC4 has the largest specific capacitance owing to its high specific surface area and hierarchical pore structure. The CV curves for SSAC*n* displayed analogous quasi-rectangular shapes at 5 mV s^−1^ (Fig. 8b), indicating a typical electric double-layer capacitive behavior. In addition, SSAC4 showed the largest CV curve area, further indicating that SSAC4 has the largest capacitance value.

**Fig. 9:**
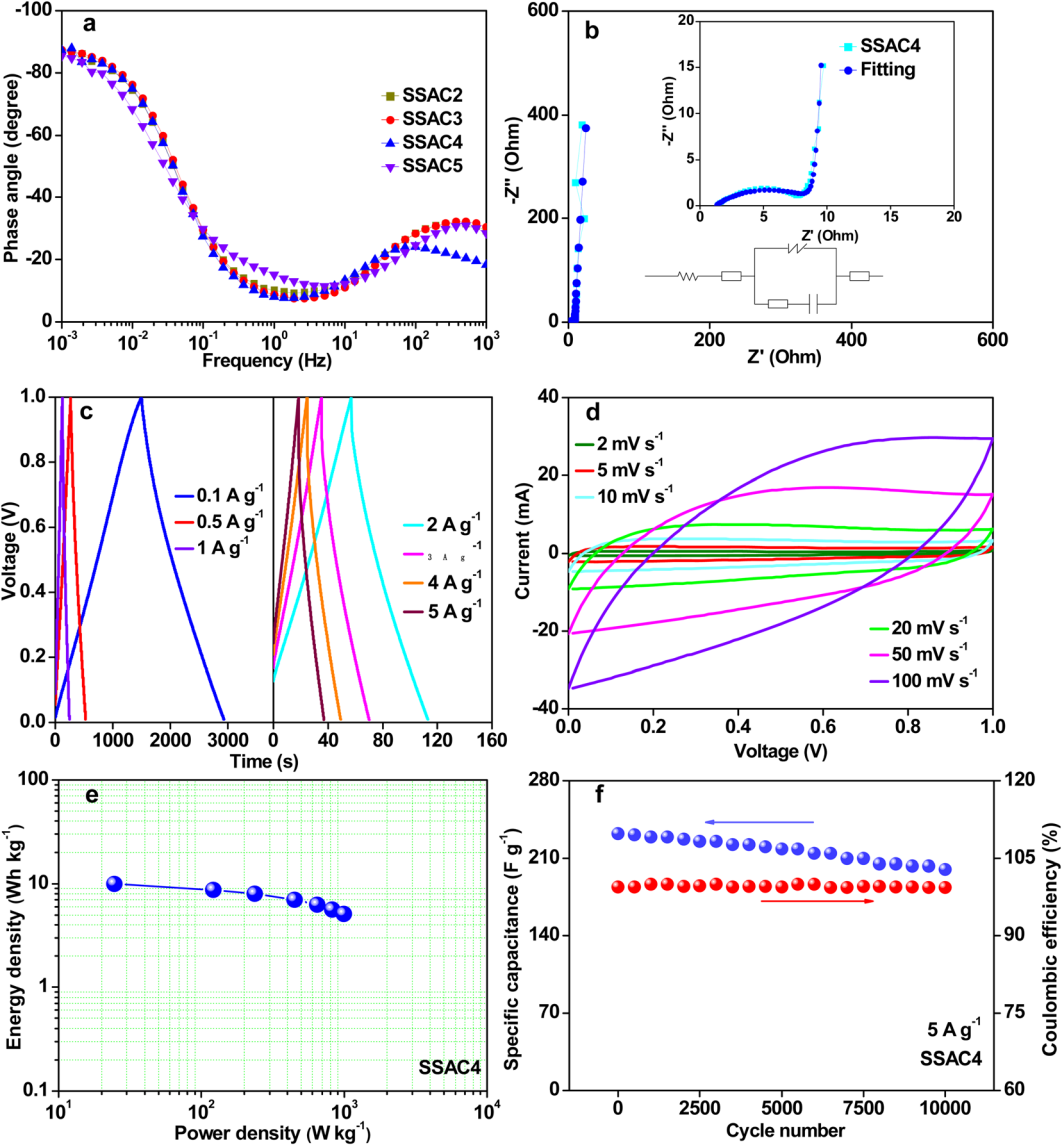
The Bode plot of SSAC*n* (a), Nyquist plots (insert is the equivalent circuit model) (b), GCD curves at current densities from 0.1 to 5 A g^−1^ (c), CV curves at scan rates from 2 to 100 mV s^−1^ (d), Ragone plot (e) and cycling stability at a current density of 5 A g^−1^ for 10 000 cycles (f) of the as-synthesized SSAC4 measured in all-solid-state symmetric supercapacitors with PVA/KOH as the electrolyte.

The specific capacitances of SSAC*n* at various current densities are summarized in Fig. 8c. The specific capacitances of SSAC4 are much higher than those of the other samples. Such as the specific capacitance of SSAC4 was 294 F g^−1^ at 0.1 A g^−1^, which was higher than that of SSAC2 (219 F g^−1^), SSAC3 (240 F g^−1^), and SSAC5 (237 F g^−1^). When the current density was increased by 50 times, the specific capacitance of SSAC4 was still maintained at 232 F g^−1^, indicating the good rate capability of SSAC4. The Nyquist plots of SSAC*n* are displayed in Fig. 8d. It can be clearly seen that the SSAC*n* possesses nearly vertical lines at low frequency, suggesting the characteristic behavior of the ideal capacitive and low ion diffusion resistance.^37^ The result can also be obtained from Fig. 9a. The phase angles of SSAC*n* are close to −90°, indicating the ideal capacitive behavior. Compared with the other three samples (SSAC2, SSAC3, and SSAC4), SSAC5 has a high mesopores-specific surface area and more mesopores, which results in the low *R*_ct_ of SSAC5. The EIS experimental result of SSAC4 was simulated by the equivalent circuit model and the result is presented in Fig. 9b. The capacitor circuit was composed of *R*_s_, *W*, *R*_ct_, *C*_1_, and *C*_2_, where *R*_s_ is the sum of the contact resistance and material resistance, *W* is the Warburg resistance, *R*_ct_ is the charge-transfer resistance, *C*_1_ is the double-layer capacitance and *C*_2_ is the pseudocapacitance produced by a heteroatom. The simulation data are in good agreement with the experimental Nyquist plots, demonstrating that the equivalent circuit model applied in this work is correct.

The GCD curves (Fig. 9c) of SSAC4 have a symmetric triangle shape from 0.1 to 5 A g^−1^, indicating the good coulomb efficiency and superior charge–discharge reversibility of SSAC4. The CV curves of SSAC4 at different scan rates are shown in Fig. 9d. All the curves display quasi-rectangular shapes even at 100 mV s^−1^, further suggesting the good rate characteristic and rapid charge transfer rate of SSAC4. The Ragone plot of SSAC4 is shown in Fig. 9e and the energy density reaches up to 9.9 W h kg^−1^ at a power density of 0.024 kW kg^−1^. The cycling stability and reversibility of supercapacitors are also important indicators for evaluating the quality of supercapacitors. As shown in Fig. 9f, the SSAC4-based supercapacitors show good specific capacitance retention (86.2%) after 10 000 cycles at a current density of 5 A g^−1^.

These results demonstrate that the SSAC4 as the electrode material for supercapacitors shows superior electrochemical performance. The reasons for the high-performance supercapacitors are as follows: (i) SSAC4 has a high specific surface area, which can provide more active sites to accommodate more charges, (ii) the high content heteroatom of O not only offers the pseudocapacitance but also enhances the wettability of the electrode surface.

## 4. Conclusions

In summary, micro-tube morphology structure porous carbon was successfully synthesized by facile carbonization followed by the KOH activation method using soybean shells as the biomass precursor. The obtained porous carbon presents a high specific surface area of 2498 m^2^ g^−1^ with a pore volume of 1.36 cm^3^ g^−1^. Due to the hierarchical pore structure, high specific surface area, and doping of heteroatoms, the SSAC4 electrode displays an ultrahigh specific capacitance of 465 F g^−1^ at a current density of 1 A g^−1^ in a three-electrode system with 6 M KOH electrolyte. Furthermore, the assembled SSAC4//SSAC4 all-solid-state symmetric supercapacitors delivered a high specific capacitance of 294 F g^−1^ at 0.1 A g^−1^, as well as excellent cycling stability of 86.2% after 10 000 cycles at 5 A g^−1^.

## Conflicts of interest

We declare that we do not have any commercial or associative interests that may represent a conflict of interest in connection with the work submitted.

## Supplementary Material
